# Combination therapy of menstrual derived mesenchymal stem cells and antibiotics ameliorates survival in sepsis

**DOI:** 10.1186/s13287-015-0192-0

**Published:** 2015-10-16

**Authors:** Francisca Alcayaga-Miranda, Jimena Cuenca, Aldo Martin, Luis Contreras, Fernando E. Figueroa, Maroun Khoury

**Affiliations:** Laboratory of Nano-Regenerative Medicine, Faculty of Medicine, Universidad de Los Andes, Santiago, 7620001 Chile; Cells for Cells, Santiago, 7620001 Chile; Clínica Universidad de Los Andes, Santiago, 7620001 Chile; Consorcio Regenero, Santiago, 7620001 Chile

**Keywords:** Sepsis, Mesenchymal stems cells, Menstrual stem cells, MenSCs

## Abstract

**Introduction:**

Sepsis is a clinical syndrome associated with a severe systemic inflammation induced by infection. Although different anti-microbial drugs have been used as treatments, morbidity and mortality rates remain high. Mesenchymal stem cells (MSCs) derived from the bone marrow have demonstrated a partial protective effect in sepsis. Menstrual derived MSCs (MenSCs) emerge as an attractive candidate because they present important advantages over other sources, including improved proliferation rates and paracrine response under specific stress conditions. Here, we evaluate their therapeutic effect in a polymicrobial severe sepsis model.

**Methods:**

The antimicrobial activity of MenSCs was determined *in vitro* through direct and indirect bacterial growth assays and the measurement of the expression levels of different antimicrobial peptides (AMPs) by quantitative reverse transcription-polymerase chain reaction. The therapeutic effect of MenSCs was determined in the cecal ligation and puncture (CLP) mouse model. Mice were then treated with antibiotics (AB) or MenSCs alone or in combination. The survival rates and histological and biochemical parameters were evaluated, and the systemic levels of pro- and anti-inflammatory cytokines as well as the response of specific lymphocyte subsets were determined by flow cytometry.

**Results:**

MenSCs exerted an important antimicrobial effect *in vitro*, mediated by a higher expression of the AMP-hepcidin. In the CLP mouse model, MenSCs in synergy with AB (a) improved the survival rate (95 %) in comparison with saline (6 %), AB (73 %), and MenSCs alone (48 %) groups; (b) enhanced bacterial clearance in the peritoneal fluids and blood; (c) reduced organ injuries evaluated by lower concentrations of the liver enzymes alanine aminotransferase and aspartate aminotransferase; and (d) modulated the inflammatory response through reduction of pro- and anti-inflammatory cytokines without significant loss of T and B lymphocytes.

**Conclusions:**

We conclude that MenSCs in combination with AB enhance survival in CLP-induced sepsis by acting on multiples targets. MenSCs thus constitute a feasible approach for the future clinical treatment of sepsis.

**Electronic supplementary material:**

The online version of this article (doi:10.1186/s13287-015-0192-0) contains supplementary material, which is available to authorized users.

## Introduction

Sepsis is a severe medical condition which ranks among the top 10 causes of death worldwide and has permanently high incidence rates [1, 2]. Sepsis is caused by an infection and involves complex interactions between the pathogenic agent and the host immune cells, characterized by a systemic inflammatory state [3]. Although the role of the immune response is crucial to fight infection, it is also responsible for the inflammatory tissue infiltration and severe organ damage, both hallmarks of sepsis [4]. Recent evidence suggests that the modulation of pro- and anti-inflammatory factors contributes to the suppression of immune effector cells that induce the systemic inflammatory response and tissue damage in sepsis [3, 5, 6].

Mesenchymal stem cells (MSCs) are a subpopulation of multipotent cells that may be isolated from various adult tissues and organs [7]. In recent years, MSCs have been described as a novel therapeutic strategy for the treatment of diseases related with inflammation and tissue injury because they are potent modulators of immune system with the ability to regulate both the innate and adaptive immune response [1, 8, 9]. Several studies demonstrate that the protective role of MSCs in sepsis may be attributed essentially to the soluble paracrine factors released by these cells, such as interleukin-10 (IL-10) [10], prostaglandin E_2_ (PGE_2_) [3], tumor necrosis factor-alpha (TNF-α)-stimulated gene/protein 6 [11], and IL-6 [12]. In addition, another study ascribes the therapeutic effect of MSCs in sepsis to direct anti-bacterial activity mediated by the secretion of the antimicrobial peptide LL37/hCAP18 [13]. In all preclinical models, therapy with MSCs showed important therapeutic advantages associated with an increase in survival time, a decrease in the levels of biomarkers associated with organ injury and the bacterial loads in different organs and biological fluids, and modulation of the immune response [3, 10, 14–16].

Currently, most studies related to MSC therapy in inflammatory and tissue damage have focused on MSCs derived from bone marrow (BMSCs) or adipose tissue (AT-MSCs) [5, 10, 13, 15, 16], mainly because these two sources of MSCs have been well characterized and share immunomodulatory properties [16, 17]. However, from a clinical point of view, large amounts of MSCs must be isolated and expanded at low culture passages to obtain the required number of cells for therapeutic application. It should also be considered that in some cases multiple administrations are necessary to reach therapeutic effect. Because of this, the study of new sources of MSCs resolving these issues could be an important advantage for the treatment of sepsis, and the comparison between MSCs from different tissues will contribute to select the best source for this application. In this regard, MSCs derived from menstrual fluid (MenSCs) emerge as an attractive alternative of cell therapy since they are isolated in a non-invasive manner with the possibility of periodical collections from the same donor, ensuring high amounts of cells at low culture passages and from the same genetic background [18, 19].

In the present study, we assessed the therapeutic effect of MenSC therapy in an animal model of severe sepsis induced by cecal ligation and puncture (CLP), the main model for polymicrobial human sepsis [20]. We evaluated the antimicrobial activity of MenSCs *in vitro* and assessed the expression of different antimicrobial peptides (AMPs) that could be involved in the anti-bacterial effect. In addition, we studied the capacity of the MenSCs to reduce the systemic inflammatory state and organ dysfunction through the modulation of the immune response and expression of tissue protective/regenerative factors. Moreover, we compared the therapeutic effect of MenSCs and their synergy with antibiotic treatment, considered as the first-line therapy for sepsis.

## Methods

### Menstrual fluid and bone marrow samples

After informed consent from the donors and ethical revision and approval from the ethics committees of Universidad de los Andes and Cells for Cells, menstrual fluids were collected from four healthy donors from 24 to 38 years old and bone marrow from three hip surgery patients from 60 to 72 years to old as described previously [19].

### Cell culture

MSCs were isolated as described previously [19]. In brief, MSCs were cultured in Dulbecco’s modified Eagle’s medium (DMEM) (Invitrogen, part of Thermo Fisher Scientific, Waltham, MA, USA) supplemented with 10 % fetal bovine serum (FBS) (Gibco, Carlsbad, CA, USA), 1 % penicillin–streptomycin (Invitrogen), and 1 % L-glutamine (Invitrogen). Cells were cultured at 37 °C with 5 % CO_2_, routinely tested for mycoplasma (EZ-PCR Mycoplasma test kit; Biological Industries, Israel Beit-Haemek Ltd, Kibbutz Beit-Haemek, Israel) and cryopreserved in low passage (<3) until use. All cells were evaluated in their capacity to differentiate to adipocytes, osteocytes, and chondrocytes by using the StemPro Differentiation Kits (Gibco, Carlsbad, CA, USA) in accordance with the instruction of the manufacturer. Immunophenotyping was performed by fluorescence-activated cell sorting (FACS) by using a FACSCanto II cytometer (BD Biosciences, San Jose, CA, USA) after staining with monoclonal antibodies CD105, CD90, CD73, CD44, HLA-DR, CD34, and CD45 (all from BD Pharmingen, San Jose, CA, USA) by using standard protocol. All experiments were performed by using cells in passage 4–8.

### Antimicrobial assay

Assessments of bacterial growth inhibition were performed as direct and indirect assays. Bacteria were isolated from mouse feces. For this, freshly harvested fecal material was diluted in 10 ml of sterile phosphate-buffered saline (PBS) and filtered through a 70-μm Cell Strainer (BD Falcon, Franklin Lakes, NJ, USA). Colony-forming unit (CFU) concentration was calculated by counting colonies plated on blood-agar plates after overnight incubation at 37 °C.

For direct inhibition assays, MSCs cultured in 24-well plates (2 × 10^5^ cells per well) in DMEM supplemented with 5 % FBS and 1 % L-glutamine were infected with 300 CFUs of a mix of bacteria and incubated for 6 or 24 hours at 37 °C with 5 % CO_2_ (normoxia) or 24 hours at 37 °C with 1 % O_2_ (hypoxia). Aliquots of the culture medium were taken from each well, serially diluted with sterile PBS, and plated on blood-agar plates. Colonies were counted after overnight incubation at 37 °C. Antimicrobial activity of MSCs conditioned medium (CM) (non-stimulated CM condition) or from the co-culture above (stimulated CM condition) was tested as described previously with slight modifications [13]. Briefly, MenSCs CM and BMSCs CM were collected, and the bacterial fraction was removed by passing the CM through a 0.22-μm filter (EdLab, New York, NY, USA). The filtered CM was centrifuged at 13,200 revolutions per minute for 10 minutes, and multiple freezing and thawing cycles were then performed (to eliminate any residual bacterial contamination). Prior to the experiments, samples were thawed on ice, and aliquots of 90 μl of the CM were transferred to a 96-well plate, inoculated with 100 CFUs of bacterial mix (in 10 μl of PBS), and incubated for 16 hours at 37 °C. CFUs were then counted as described earlier.

### mRNA expression

Total RNAs was extracted by using the RNeasy kit (Qiagen, Marseille, France) in MSCs in basal condition or previously stimulated with 300 CFUs of bacterial mix. RNA (2 μg) was then reverse-transcribed by using the M-MLV enzyme (Fisher Scientific, Illkirch, France). The real-time quantitative polymerase chain reaction (PCR) was performed by using Stratagene Mx3000P (Agilent Technologies, Santa Clara, CA, USA) with the following forward (F) and reverse (R) primers: hBD-1-F primer: 5ʹ-GCCTCAGGTGGTAACTTTCTCA-3ʹ, hBD-1-R primer: 5ʹ-GATCGGGCAGGCAGAATAGAG-3ʹ; hBD-2-F: 5ʹ-GGAAGAAATGTCGCAGCCAAG-3ʹ, hBD-2-R: 5ʹ- CGATTCAGTAAGCTCTCATCCCAT-3ʹ; hBD-3-F: 5ʹ- TTATTGCAGAGTCAGAGGCGG -3ʹ, hBD-3-R: 5ʹ-TTTCTTCGGCAGCATTTTCGG-3ʹ; hepcidin-F: 5ʹ- CCCATGTTCCAGAGGCGAAG-3ʹ, hepcidin-R: 5ʹ-CTTGCAGCACATCCCACACT-3ʹ. For LL-37 peptide mRNA quantification was determined by TaqMan gene expression assay (ID Hs00189038_m1; Applied Biosystems, Waltham, MA, USA). All values were normalized to either GAPDH (GAPDH-F: 5ʹ-GGTCTCCTCTGACTTGAACA-3ʹ, GAPDH-R: 5ʹ-GTGAGGGTCTCTCTCTTCCT-3ʹ) or β2M housekeeping gene (ID Hs00984230_m1; Applied Biosystems) and expressed as relative expression or fold change using the 2^−ΔCT^ formula [21].

### Preparation of conditioned media-derived MenSCs

MenSCs were cultured in normal medium until 80 % confluence was reached. After three washes with PBS, cells were cultured in serum-free DMEM supplemented with 1 % penicillin-streptomycin and 1 % L-glutamine at 37 °C with 5 % CO_2_. After 72 hours post-culture, the supernatant was collected. The cellular debris were removed by centrifugation at 500 × *g* for 5 minutes at room temperature (RT). The supernatant was concentrated approximately 25- to 30-fold by ultrafiltration using Amicon Ultra centrifugal filters (Merck Millipore, Tullagreen, Ireland) with a membrane NMWL of 3 kDa in accordance with the instructions of the manufacturer. The concentrated medium was sterilized by filtration through a 0.22-μm syringe filter (Membrane Solutions LLC, Dallas, TX, USA), and the protein concentration was determined by Bradford protein assay (Bio-Rad Laboratories, Hercules, CA, USA). The mean protein concentration was 0.3 ± 0.2 μg/ul. Equal volumes of serum-free DMEM but without cells were used under the same conditions and served as negative control.

### Polymicrobial CLP-induced sepsis

*In vivo* studies were performed at the Universidad de los Andes-Cells for Cells Animal Facility (Santiago, Chile) in accordance with the institutional guidelines for the care and use of laboratory animals in research, revised and approved by the Institutional Ethical Committee for animal experimentation. C57BL6/j mice (8 to 12 weeks old; Jackson Laboratories, Bar Harbor, ME, USA) received *ad libitum* access to food and water during the whole experimental procedure. CLP-induced sepsis was performed as described previously [20] with some modifications. Briefly, mice were anesthetized with inhaled Sevofluorane (Baxter, Guayama, Puerto Rico) by using an anesthetic vaporizer. After a 1-cm incision was performed, the cecum was exposed and ligated in the middle portion with 5–0 dafilon suture (Braun, Rubi, Spain), punctured once with an 18-gauge needle (in order to induce high-grade sepsis), and returned to the peritoneal cavity. The abdominal incision was sutured with catgut 5–0 (Braun, Bogota, Colombia), and the skin was closed with Histoacryl (Braun). In sham-operated mice, the cecum was exposed and ligated but not punctured. Immediately after the CLP procedure, all animals received pre-warmed fluid resuscitation with 50 ml/kg saline fluid by subcutaneous injection.

### Treatment of CLP-induced sepsis mice with MSCs and CM-derived MSCs

Three hours after CLP-induced sepsis, mice were randomized and distributed into different experimental groups to receive single injections of saline (vehicle) (n = 16), antibiotics (AB) (n = 22), MenSCs (n = 21), MenSCs + AB (n = 22), MenSC CM (n = 16), or MenSC CM + AB (n = 22). MenSCs (7.5 × 10^5^ cells/mice) and MenSC CM (70 μg/300 μl) were inoculated via intra-peritoneal or intra-venous injection, respectively, alone or with Enrofloxacin 5 mg/kg (Centrovet, Santiago, Chile). For Kaplan-Meier survival curves, animals were assessed after CLP-induced sepsis every 12 hours for 4 days. Fluid resuscitation and antibiotic therapy were administered by subcutaneous injections immediately after the CLP procedure and repeated every 24 hours for 4 days. For the evaluation of the therapeutic effect, animals were sacrificed at 24–40 hours after CLP-induced sepsis, and total blood was collected by cardiac puncture for measurement of hematological and biochemical parameters and cytokine levels. The peritoneal cavity was washed, and lavage fluid was collected to determine the bacterial CFUs as previously described [22]. Liver, kidney, and lungs were removed, fixed in 10 % formalin solution, and embedded in paraffin by standard methods. Samples were cut into 5-μm sections and stained with hematoxylin and eosin in accordance with standard protocols.

### Biodistribution of MenSCs injected in septic mice

At 24 hours post-CLP procedures, 10^7^ MenSCs were labeled with PKH26 (Sigma-Aldrich, St. Louis, MO, USA) in accordance with the protocol of the manufacturer. Labeling efficiency was 95 % as validated by flow cytometry. Labelled MenSCs (2 × 10^6^ cells/mouse) were resuspended in 250 μl of PBS and injected intraperitoneally. Animals were euthanized at 24 hours post-injection, and different organs and fluids, including the spleen, heart, kidneys, lungs, liver, and intraperitoneal fluid, were recovered and incubated at 37 °C for 30 minutes with 250 U/ml of collagenase II and IV (Gibco). The single-cell suspensions obtained were resuspended into 400 μl of cytometry buffer (PBS 1×, bovine serum albumin (BSA) 0.2 %, sodium azide 0.01 %) and then analyzed by flow cytometry.

### Bacterial numbers in blood, peritoneal cavity, and spleen in CLP-induced sepsis

Total blood, peritoneal fluid, and homogenized spleen in sterile saline were plated in a 10-fold dilution on blood-agar plates and incubated at 37 °C overnight, and the number of CFUs was determined.

### Cytokine multiplex array evaluation in CLP mice

The levels of inflammatory cytokines—monocyte chemoattractant protein-1 (MCP-1), TNF-α, IL-6, and IL-8—in CLP mice serum samples were determined by using a commercial BD™ Cytometric Bead Array Mouse Inflammation Kit (BD Biosciences Pharmingen, San Diego, CA, USA) in accordance with the instructions of the manufacturer. Analysis was carried out by flow cytometry, and the concentration (in picograms per milliliter) was determined by using FCAP Array software (BD Biosciences). The parameters were determined 24 and 40 hours after CLP surgery.

### Blood biochemical analysis in CLP mice

Plasma levels of blood urea nitrogen (BUN), bilirubin, alanine aminotransferase (ALT), aspartate aminotransferase (AST), alkaline phosphatase (ALP), albumin, amylase, and glucose were evaluated by using the Piccolo Xpress™ Chemistry Analyzer (Abaxis, Union City, CA, USA) in accordance with the instructions of the manufacturer. Serum creatinine was determined by using a commercial kit (Valtek Diagnostics, Santiago, Chile) in accordance with the instructions of the manufacturer.

### Lymphocyte subset analysis by FACS

Blood was collected by intracardiac puncture with EDTA as anticoagulant. The red blood cells were lysed with ACK Lysing Buffer (Gibco) in accordance with the instructions of the manufacturer. The collected cells were resuspended in 100 μl of FACS buffer (PBS 1×, 0.2 % BSA, 0.01 % sodium azide) and incubated for 20 minutes at 4 °C with the appropriate fluorescently labeled monoclonal antibody directed against lymphocyte surface markers (BD Biosciences), washed and resuspended in FACS buffer, and analyzed by the FACS Canto II cytometer by using the FACS Diva software (BD Biosciences). The viability was determined by using a LIVE/DEAD®Fixable dead cell stain kit (Invitrogen) in accordance with the protocol of the manufacturer. Approximately 20,000 gated events were assessed to determine the percentage of each subset: total lymphocytes (CD3^+^/APC), CD4^+^ T lymphocytes (CD3^+^ APC, CD4^+^/PE), CD8^+^ T lymphocytes (CD3^+^/APC, CD8^+^/FITC), and total B lymphocytes (CD19^+^/FITC). The CD4/CD8 and CD3/CD19 ratios were calculated as the percentage of CD4^+^ T cells divided by the percentage CD8^+^ T cells and the percentage of CD3^+^ T cells divided by the percentage CD19^+^ T cells.

### Statistical analysis

Data are expressed as mean ± standard error. Comparisons of mortality were made by analyzing Kaplan-Meier survival curves and then log-rank test to assess for differences in survival. Mann-Whitney *U* test was used to evaluate the differences between groups. One-way analysis of variance followed by Tukey’s post-test was used for analysis of multiple comparison groups. The numbers of samples per group (n) are specified in the figure legends. Statistical significance was set at *P < 0.05, **P < 0.01, and ***P < 0.001.

## Results

All MSCs used in this study showed adherence to plastic, expression of classic MSCs markers, and differentiation to osteoblasts, adipocytes, and chondroblasts (Additional file 1: Figure S1) in accordance with previously described criteria [19].

### MenSCs exert anti-microbial activity mediated by the secretion of hepcidin

**Fig. 1:**
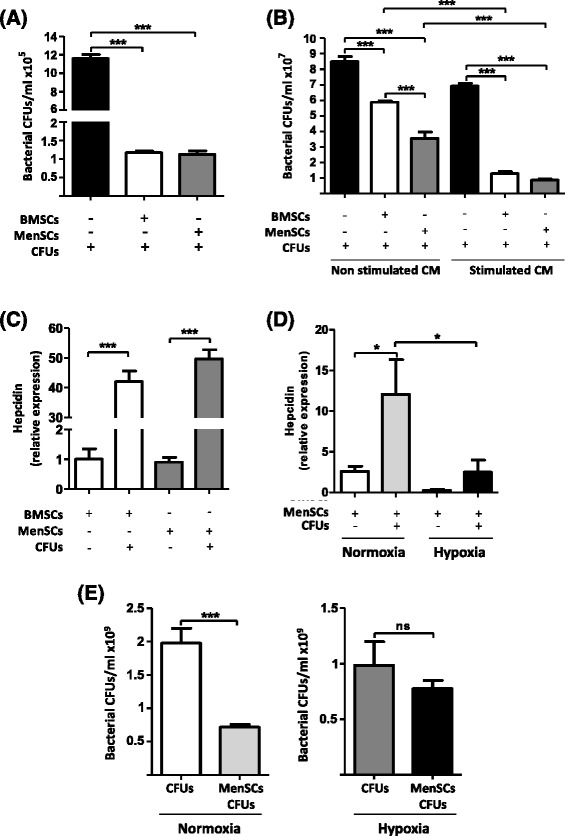
MenSCs exert an important *in vitro* anti-microbial effect, which is associated in part with the increased expression of hepcidin. MenSCs and BMSCs were evaluated in their capacity to inhibit bacterial proliferation. **a** Direct inhibition of the bacterial proliferation. Cells were cultured in direct contact with a bacterial mixture. After 6 hours, the CM was collected and plated on blood-agar plates. Values are expressed as CFUs per milliliter. **b** Indirect inhibition of bacterial proliferation. CM of cells cultured with or without bacterial stimulation was collected and their antimicrobial activity was tested by the incubation with a bacterial mixture. Values are expressed as CFUs per milliliter. **c** Relative expression of hepcidin. The mRNA expression levels of hepcidin under both basal and stimulated conditions are determined by qRT-PCR. **d** Inhibition of hepcidin by hypoxia. The mRNA expression levels of hepcidin under normoxic and hypoxic culture conditions in basal and stimulated MenSCs are determined by qRT-PCR. **e** Direct inhibition of the bacterial proliferation under hypoxic conditions. MenSCs were cultured in direct contact with a bacterial mixture under normoxic and hypoxic conditions for 24 hours. After 24 hours, the CM was collected and plated on blood-agar plates. Values are expressed as CFUs per milliliter. Data are presented as the mean ± standard error. *BMSCs* bone marrow-derived mesenchymal stem cell*s*, *CFU* colony-forming unit, *CM* conditioned medium, *MenSCs* menstrual derived mesenchymal stem cells, *ns* non-significant, *qRT-PCR* quantitative reverse transcription-polymerase chain reaction

*In vitro* assays were performed to evaluate the direct and indirect anti-microbial MSC activity on bacterial growth. Since the anti-bacterial properties of BMSCs are well known, we decided to compare the anti-microbial effect of MenSCs with respect to BMSCs to establish the comparative anti-microbial potency of MenSCs. For the direct assay, MSCs were incubated with a bacterial mixture for 6 hours. As shown in Fig. 1a, both MenSCs and BMSCs exerted a significant inhibition of the bacterial growth in comparison with control (*P* ≤ 0.001). To determine whether this anti-bacterial effect is associated with the secretion of soluble factors, CM was studied (indirect assay) in accordance with published protocols [13]. Whereas both non-stimulated and stimulated CM exhibited important anti-bacterial activity, MenSCs non-stimulated CM showed a greater inhibition of bacterial growth compared with BMSCs non-stimulated CM (*P* ≤ 0.001) (Fig. 1b). This effect was enhanced when CM was collected from MSCs previously stimulated with a bacterial load prior to incubation for direct assays (*P* ≤ 0.001). Taken together, these data suggest that inhibition of bacterial growth is associated with the secretion of soluble factors by MSCs and that, at least in *in vitro* conditions, MenSCs appear to be a better cell candidate for control of bacterial growth compared with BMSCs.

To better understand the anti-microbial activity exhibited by MenSCs, we analyzed the expression of different AMPs under basal conditions and after bacterial stimulation. Levels of LL37 and β-defensin (hBD-1, -2, and -3) could not be detected in MenSCs and BMSCs in either non-stimulated or stimulated conditions (data not shown). Interestingly, a low expression level of hepcidin was detected in both cell sources. The bacterial stimulation induced the increase of hepcidin expression up to 42- and 50-fold in BMSCs and MenSCs, respectively (*P* ≤ 0.001) (Fig. 1c). Hypoxic culture conditions are known to inhibit the expression of hepcidin in hepatoma cells [23]. Therefore, MenSCs were cultured for 24 hours in both normal and hypoxic conditions and in the presence or absence of the bacterial stimulus. Under hypoxia, the expression of hepcidin decreased in both conditions; however, statistical significance was obtained only for the stimulated MenSCs (*P* ≤ 0.05), abrogating the effect of bacterial stimulus on hepcidin expression (Fig. 1d). Furthermore, the inhibition of hepcidin by the hypoxic conditions entailed a loss of the anti-microbial activity of MenSCs in comparison with normoxic conditions (Fig. 1e).

### MenSCs improve survival and multiorgan dysfunction in the CLP-induced sepsis model

**Fig. 2:**
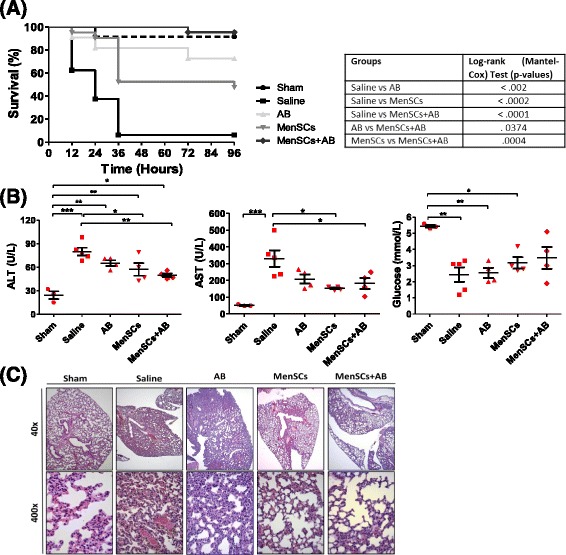
Treatment with MenSCs alone or in combination with antibiotics improves survival and protects against multiorgan dysfunction in CLP-induced sepsis. **a** Survival curves in mice with polymicrobial sepsis and different treatments. C57BL6/J mice subjected to CLP-induced sepsis were randomized into five groups: sham (n = 12), saline (n = 16), antibiotics (AB) (n = 22), MenSCs (n = 21), and MenSCs + AB (n = 22). Three hours post-surgery, mice were injected with saline (vehicle), AB and/or MenSCs. Survival percentages of untreated and treated mice after CLP are presented as a Kaplan-Meier survival curve. **b** Serum concentration of alanine aminotransferase (ALT), aspartate aminotransferase (AST), and glucose after CLP-induced sepsis. Serum was isolated 24 hours post-surgery and treatment administration (sham, n = 3; saline, n = 5; AB, n = 4; MenSCs, n = 4; MenSCs + AB, n = 4), and the concentrations of biomarkers of liver function and total glucose were determined. Dot plots represent individual values, horizontal bars represent mean values, and vertical bars represent standard error values. **c** Lung histology after CLP-induced sepsis. At 40 hours post-surgery and treatment infusion, lungs were collected, fixed, embedded in paraffin, and stained with hematoxylin-eosin. Pictures are representative images of lungs from the different experimental groups. *AB* antibiotics, *CLP* cecal ligation puncture, *MenSCs* menstrual derived mesenchymal stem cells

Because MenSCs showed higher bacterial inhibition potential *in vitro*, their therapeutic effect in sepsis was evaluated by using a CLP-induced sepsis model (Additional file 2: Figure S2). At 96 hours after different treatment, a significant increase in the survival rate of MenSCs + AB treatment group was observed in comparison with all the other experimental groups (saline, *P* ≤ 0.0001; AB, *P* = 0.0374; MenSCs, *P* = 0.0004) as shown in Fig. 2a. The survival rate reached by MenSCs + AB group was 95 % (21/22), while survival in the saline, AB, and MenSCs groups was 6 % (1/16), 73 % (16/22) and 48 % (11/21), respectively. Both conventional AB and MenSCs treatments increased the survival of animals compared with saline (*P* ≤ 0.002 and *P* ≤ 0.0002, respectively). No differences were observed between AB group compared with the MenSCs group.

Because sepsis lethality is associated with multiple organ failure, biochemical indicators of hepatic, renal, and pancreatic function were assessed in serum samples. No differences were observed between the different groups in the study with respect to renal functional profile (serum creatinine and BUN levels) and pancreatic functional marker (amylase levels) (data not shown). However, liver function was markedly improved in animals that received MenSCs without or with AB (Fig. 2b). The concentrations of the liver enzymes ALT and AST were detected at lower values in the MenSCs (ALT 57.25 ± 8.107 U/l *P* ≤ 0.05; and AST 153.5 ± 5.299 U/l *P* ≤ 0.05) and MenSCs + AB group (ALT 49.75 ± 2.428 U/l *P* ≤ 0.01; and AST 182.3 ± 1.99 U/l *P* ≤ 0.05) compared with the saline group (ALT 79.8 ± 5.083 U/l and AST 331.2 ± 49.29 U/l), suggesting that the protective or regenerative liver effect seems to be contributed mainly by the MenSCs. Although no difference was found in the concentration of ALP enzyme, the analysis showed similar concentrations between sham (47.67 ± 3.18 U/l), MenSCs (48.75 ± 5.17 U/l), and MenSCs + AB (44.75 ± 5.45 U/l) groups, in contrast to the increased levels observed in saline (90 ± 21.47 U/l) and AB (74.25 ± 12.03 U/l) groups (Additional file 3: Figure S3). Altered blood glucose concentration is commonly observed in sepsis and is associated with adverse outcome [24]. Decreased serum albumin levels are associated with acute-phase response of the liver [25, 26] and an increased capillary leakage [27, 28]. After MenSCs + AB treatment, we observed a normalization in the blood glucose and albumin levels, indicating a recovery in the organ-wide metabolic failure. Specifically, animals from the MenSCs + AB group (3.475 ± 0.6787 U/l) exhibited levels similar to animals from the sham group (5.433 ± 0.088 U/l), but animals from the saline (2.44 ± 0.45 U/l), AB (2.55 ± 0.31 U/l) and MenSCs (3.175 ± 0.35 U/l) groups had a decrease in serum glucose concentration (Fig. 2b). The levels of serum albumin demonstrated a reduction in the saline group (2.440 ± 0.4490 g/dl) compared with the sham group (5.433 ± 0.088 g/dl *P* ≤ 0.01), but an upward tendency was observed in groups of animals that received cells (Additional file 3: Figure S3).

Although the histological examination of kidney and liver at 40 hours post-surgery showed no significant differences among groups (data not shown), consistently with other reports [5, 29] lung histology assessment revealed that MSCs treatment effectively prevented acute lung injury (Fig. 2c). In lungs of animals from the saline group, vast areas of atelectasis were observed with marked venular and capillary congestion with reduced airspaces and a moderate amount of septal leucocytes. In contrast, in all treated groups, the lungs showed irregular distended alveoli and isolated areas of atelectasis with moderate capillary congestion, irregular alveolar distension, and normal amounts of septal leukocytes.

### MenSC treatment downregulates the inflammatory responses *in vivo*

**Fig. 3:**
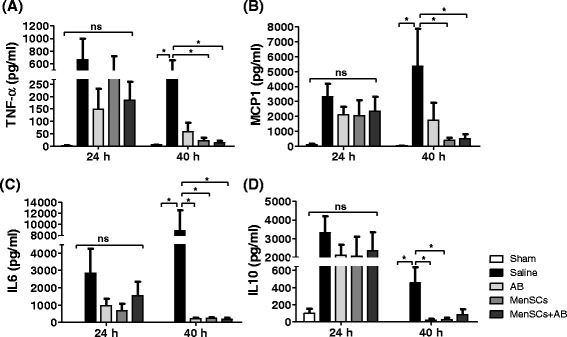
MenSCs modulate the response of the host immune system to sepsis. At 24 and 40 hours after CLP-induced sepsis and treatment administration, blood samples were obtained to determine the serum concentration of the inflammatory cytokines **(a)** TNF-α, **(b)** MCP-1, **(c)** IL-6, and **(d)** IL-10 using a Cytometric Bead Array and analyzed by flow cytometry (sham, n = 6; saline, n = 3–11; AB, n = 4–6; MenSCs, n = 5–6; MenSCs + AB, n = 6). Histograms represent the mean ± standard error. *AB* antibiotics, *CLP* cecal ligation and puncture, *IL* interleukin, *MCP-1* monocyte chemoattractant protein-1, *MenSC* menstrual derived mesenchymal stem cells, *ns* non-significant, *TNF-α* tumor necrosis factor-alpha

To determine the effect of MenSCs on the immune response to infection, serum TNF-α, IL-6, MCP-1, and IL-10 were determined. Whereas at 24 hours post-CLP no differences in the serum levels of cytokines were detected, at 40 hours a clear modulation of the inflammatory response was observed in treated animals (Fig. 3). Specifically, a reduction in the TNF-α and MCP-1 serum concentration was observed in the MenSCs with and without AB groups in comparison with the saline group (*P* ≤ 0.05) (Fig. 3a, b). Also, IL-6 serum levels were decreased in the AB, MenSCs, and MenSCs + AB groups in comparison with the saline group (*P* ≤ 0.05) (Fig. 3c). Antibiotic and MenSCs groups showed a reduction in the levels of IL-10 in comparison with untreated mice (*P* ≤ 0.05) (Fig. 3d). Taken together, our data suggest that while animals receiving treatment showed modulation of the innate immune response, only groups where MenSCs without or with AB presented a significant reduction in pro- and anti-inflammatory cytokines, suggesting that MenSCs can effectively rescue mice from the uncontrolled systemic inflammatory response after CLP-induced sepsis.

**Fig. 4:**
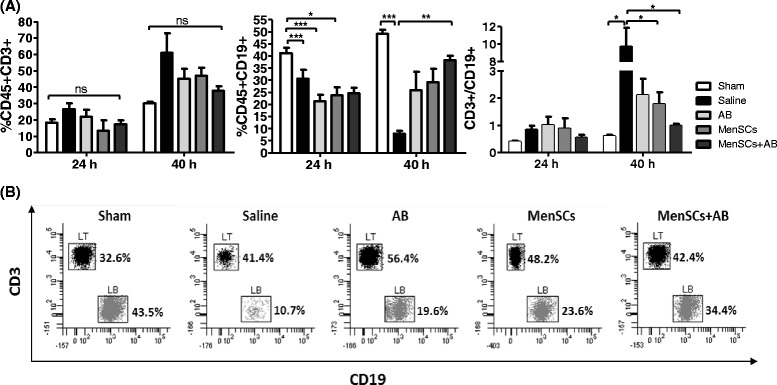
MenSCs in combination with antibiotics prevent the decrease of CD45^+^ CD3^+^ and CD45^+^ CD19^+^ lymphocyte levels after CLP-induced sepsis. Blood samples were obtained 24 and 40 hours after administration of various treatments post-CLP, and specific lymphocyte subsets were determined by fluorescence-activated cell sorting. **a** Graphs represent the percentage of CD45^+^ CD3^+^ and CD45^+^ CD19^+^ lymphocytes and the ratio CD45^+^ CD3^+^/CD45^+^ CD19 (sham, n = 3–6; saline, n = 3–10; AB, n = 3–6; MenSCs, n = 3–6; MenSCs + AB, n = 3–6). Histograms represent the mean ± standard error. **b** Representative dot plots of specific lymphocyte subsets described in (a) at 40 hours post-induction of sepsis and administration of different treatments. *AB* antibiotics, *CLP* cecal ligation and puncture, *LB* B lymphocytes, *LT* T lymphocytes, *MenSCs* menstrual derived mesenchymal stem cells, *ns* non-significant

With the progression of sepsis, the adaptive immune system reduces their efficiency by an increase in anti-inflammatory mediators and loss of T and B cells via apoptosis [30, 31]. At 24 hours, increases of the distribution of CD45^+^ CD3^+^ CD8^+^ and CD4^+^/CD8^+^ were observed in the MenSCs group in comparison with the saline group (*P* ≤ 0.05); however, at 40 hours, no differences were detected between the experimental groups (Additional file 4: Figure S4). In CD45^+^ CD3^+^ cell populations, no difference between groups was detected at 24 and 40 hours post-CLP. However, a reduction in circulating CD45^+^ CD19^+^ lymphocytes was observed at 24 hours in treated (*P* ≤ 0.05; *P* ≤ 0.001) and non-treated (*P* ≤ 0.001) groups, showing an important recovery exclusively in treated animals at 40 hours, reaching a significant increase in the MenSCs + AB group (*P* ≤ 0.01) (Fig. 4a) in comparison with untreated animals. In consequence, a reduction was also observed in the CD45^+^ CD3^+^/CD45^+^ CD19^+^ ratio in cell-treated groups in comparison with the untreated group (*P* ≤ 0.05). Representative dot plots of the specific lymphocyte subsets at 40 hours after sepsis induction and administration of different treatments are shown in Fig. 4b. Altogether, no significant loss of T and B lymphocytes was observed in all treated groups, suggesting that MenSCs can effectively modulate the immune response in animals with sepsis without a severe immunosuppression.

### MenSC treatment results in significant bacterial clearance in septic mice

**Fig. 5:**
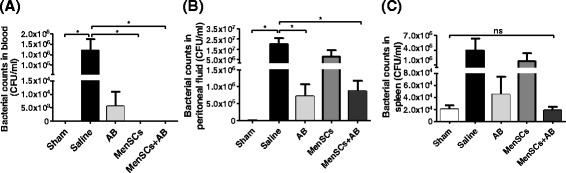
MenSCs alone or in combination with antibiotic treatment promote bacterial clearance in CLP-induced sepsis in mice. Blood, peritoneal fluid, and spleen were obtained 24 hours after administration of the different treatments (sham, n = 6; saline, n = 11; AB, n = 6; MenSCs, n = 5; MenSCs + AB, n = 6). Bacterial loads were determined after incubating at 37 °C overnight and are expressed as CFUs per milliliter. **a** Bacterial load in blood. **b** Bacterial load in peritoneal fluid. **c** Bacterial load in spleen. Data are presented as mean ± standard error. *AB* antibiotics, *CFU* colony-forming unit, *CLP* cecal ligation and puncture, *MenSCs* menstrual derived mesenchymal stem cells, *ns* non-significant

As another measure of the beneficial effect of MenSCs against infection, bacterial clearance was assessed in the blood, peritoneal fluids, and spleen of treated animals. At 24 hours post-CLP, animals that received MenSC treatment alone or in combination with antibiotics showed a lower bacterial load in blood compared with the untreated group (*P* ≤ 0.05) (Fig. 5a), reaching values similar to those of the sham group (MenSCs 0.2 ± 0.2 CFU/ml; MenSCs + AB 0.17 ± 0.17 CFU/ml; and sham 0.17 ± 0.17 CFU/ml). In peritoneal lavage, administration of AB alone or in combination with MenSCs decreased CFU counts in comparison with the saline group (*P* ≤ 0.05) (Fig. 5b). In contrast, no differences in spleen were observed among different treatment groups (Fig. 5c). Altogether, the results suggest that antibiotics seem to contribute in the control of the bacterial growth in local sepsis (abdominal cavity) but that MenSCs appear to suppress the systemic bacterial proliferation.

### Septic mice display a higher retention of MenSCs in the peritoneal cavity

**Fig. 6:**
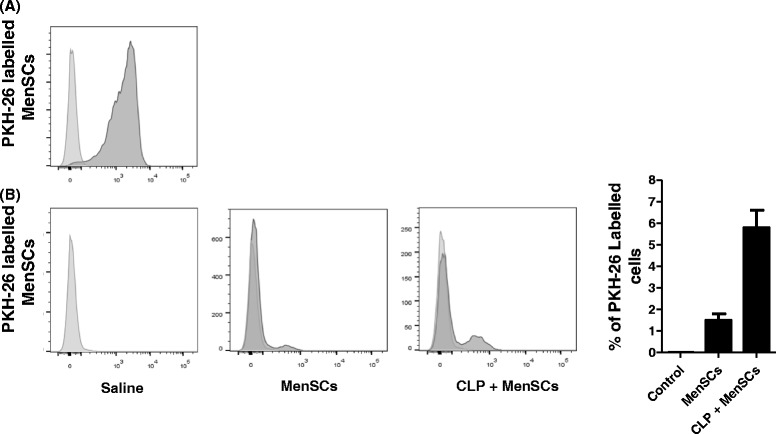
Septic mice show higher retention of the injected MenSCs in the peritoneal cavity in comparison with healthy control mice. MenSCs were labelled with PKH-26 and analyzed by flow cytometry. **a** Fluorescence-activated cell sorting plot showing a 95 % labelling efficiency. Around 2 × 10^6^ cells were injected intraperitoneally in septic or healthy mice. **b** Histogram and representative flow cytometry plots showing the percentage of remaining cells in peritoneal lavage at 24 hours post-injection. *CLP* cecal ligation puncture, *MenSCs* menstrual derived mesenchymal stem cells

To assess the fate in the peritoneal cavity of the CLP mice, MenSCs were labeled with PKH-26 (Fig. 6a) and injected intraperitoneally in healthy and sepsis-induced mice. After 24 hours post-injection, the spleen, heart, kidneys, lungs, liver, and peritoneal fluid were collected to detect labeled cells by flow cytometry analysis. Although no labeled MenSCs were detected in the different analyzed organs, the injection site contained detectable levels of PKH-26^+^ cells. Furthermore, septic mice showed a fourfold increase in the retention of injected MenSCs in the peritoneal cavity in comparison with healthy control mice (Fig. 6b).

### Conditioned media from MenSCs improve survival and multiorgan dysfunction in the CLP-induced sepsis model

**Fig. 7:**
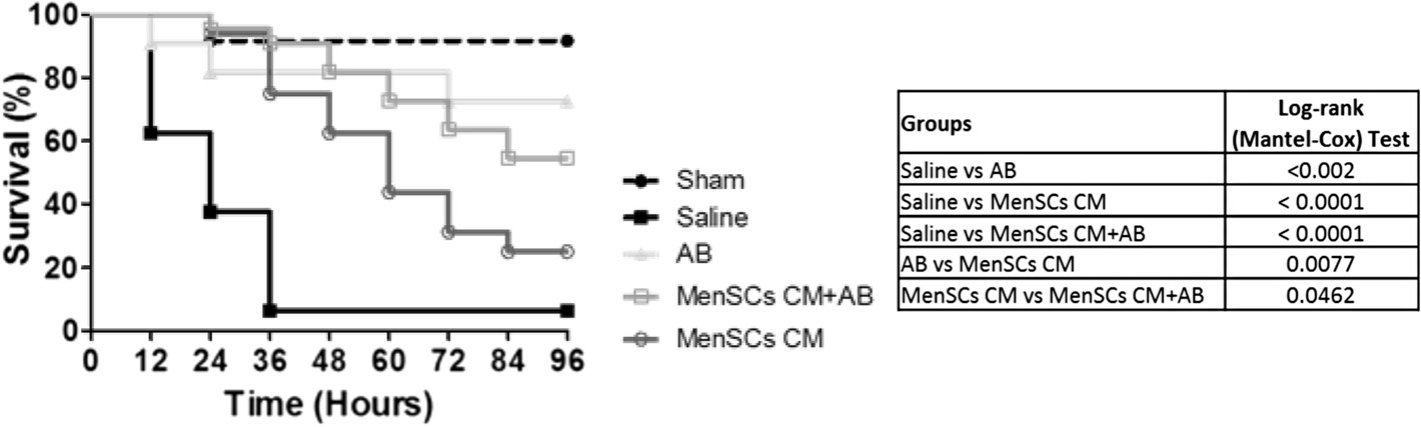
MenSCs CM improves the survival in CLP-induced sepsis in mice. C57BL6/J mice with CLP-induced sepsis were randomized in five groups: sham (n = 12), saline (n = 16), antibiotics (AB) (n = 22), MenSCs CM (n = 16), and MenSC CM + AB (n = 22). After 3 hours post-sepsis induction, mice were injected with saline (vehicle), AB and/or MenSC CM. Survival percentages of untreated and treated mice are represented as a Kaplan-Meier survival curve. *AB* antibiotics, *CFU* colony-forming unit, *CLP* cecal ligation and puncture, *CM* conditioned medium, *MenSCs* menstrual derived mesenchymal stem cells

Multiple published reports have revealed that secretion of a broad range of bioactive molecules is behind the therapeutic benefits of MSCs in sepsis. In this context and in line with the marked inhibition of bacterial growth observed *in vitro* (Fig. 1), we decided to evaluate the *in vivo* therapeutic effect of the paracrine factors secreted by MenSCs in the CLP-induced sepsis model (Fig. 7)*.* Notably, at 96 hours post-sepsis induction, MenSCs CM without or with AB increased survival compared with saline group (*P* < 0.0001). The MenSC CM + AB group exhibited a larger increase in survival than that where MenSC CM was administered alone with survival rates of 55 % (12/22) and 25 % (4/16), respectively (*P* = 0.0462). Interestingly, although the improvement in survival of the MenSCs CM + AB treatment group matched that observed in the MenSCs treatment group, it did not surpass that of the MenSCs + AB treatment.

## Discussion

MenSCs are a fully characterized multipotent stromal cell population [19, 32] that has shown several regenerative properties in different preclinical animal models [33–36]. In sepsis, the use of MSCs to combat the systemic infection [14] or modulate the response of the host immune system to the disease [3, 10] has been studied previously, showing that MSCs might be beneficial in sepsis treatment when infused at the disease onset. Because patients with sepsis show a fast deterioration of a patient’s condition in the first few hours of onset, an MSCs treatment might be administered with an identical promptness [37]. In this context, MenSCs present some advantages over MSCs derived from other sources, such as the high frequency of mesenchymal progenitors and proliferative rate [19] that allow a clinical-scale allogeneic transplantation in less time. Allogeneic MSCs therapy is regarded as a preferred source for treatments because it allows a cell infusion with a ready-to-use and off-the-shelf product in acute life-threatening indications such as sepsis, in which isolation and expansion of autologous MSCs are not options [38]. In the study presented here, we demonstrate that MenSCs in synergy with antibiotics can beneficially alleviate sepsis-associated symptoms and improve survival.

In the *in vitro* comparative study, we demonstrate that MenSCs exert an important anti-microbial effect, both directly and through factors present in its CM. In contrast with the previously published data, in which expression of the antimicrobial peptide LL-37 was detected following stimulation with *Escherichia coli* [13], we did not observe any basal or stimulated expression of this gene under both bacterial mixture and LPS stimulations (data not shown). On the other hand, we observed an elevated mRNA expression of the AMP-hepcidin after a polybacterial stimulation. Hepcidin, a peptide hormone synthesized mainly in the liver, is the principal regulator of systemic iron homeostasis that has been detected in high levels during infections and inflammation [38, 39]. To further understand the implication of hepcidin in the observed antibacterial effect, its expression was downregulated through hypoxic culture. In fact, it has been reported that hypoxia inhibited the hepcidin expression in different cell types via decreased SMAD4 signaling pathway [23, 39, 40]. We first show that hepcidin expression in MenSCs is suppressed in hypoxia. The inhibition of hepcidin results in the loss of the antimicrobial property of MenSCs, suggesting a hepcidin-dependent mechanism. Moreover, it has been demonstrated that hepatic hepcidin knockdown mice displayed a decreased bacterial clearance, increased mortality, exacerbated organ damage, and a compromised host inflammatory response following sepsis induction [40]. To our knowledge, these results provide, for the first time, evidence of hepcidin expression in MSCs and their implication in the antimicrobial effect. However, further investigation is required to look at the pathophysiological levels of hepcidin in the menstrual fluid following intra-uterine infections and also the effect of their dysregulation in this context.

In the study involving an *in vivo* animal model, MenSCs were capable of reducing animal mortality regulating different traits of sepsis, such as the organ dysfunction, modulation of the inflammatory response without severe immunosuppression, and promotion of bacterial clearance, to a degree similar to that of the standard clinical sepsis treatment (antibiotic therapy). Notably, the synergism between MenSCs + AB resulted in the highest amelioration of the survival animal proportion, indicating that the arrival of the MenSCs to the clinic should be in combination with the current therapy of sepsis. Paracrine factors have been postulated as a mechanism in the regeneration/protection of tissues in lung injury and inflammation [41], acute myocardial infarction [42, 43], and sepsis [3, 5]. In this context, we further investigated the effect of the soluble antimicrobial and other tissue protective factors secreted by MenSCs by treating animals with its CM. Although the experimental results showed prolonged survival in mice infused with MenSCs CM in comparison with the untreated group, the improvement did not match the level of survival observed in the MenSCs treated groups. This suggests that although the CM had a beneficial effect, the presence of cells in the inflammatory and infectious context is necessary to achieve the important therapeutic effect obtained. Indeed, it is well known that many MSC-expressed factors can be activated in response to stress or specific stimulation, similar to hepcidin expression following microbial stimulation. Although it is interesting to test the effect of pre-activated cells or stimulated CM in future experimental plans, the dose (low, medium, or high), frequency (one or multiple), and the time of administration (early or late) of the infusion should be defined as well.

While the present results provide a new source and evidence regarding the therapeutic effect of allogeneic MSCs in sepsis, significant advances are required to fully understand the multiple mechanisms of action behind the cell-based therapy. In addition, there are significant limitations that need to be addressed, taking into consideration the narrow time frame for the treatment of sepsis, where ready-to-inject dose of cells needs to be available.

## Conclusions

The combination of the *in vitro* and *in vivo* experiments obtained here shows that MenSCs can alleviate sepsis-associated symptoms. The mechanisms by which MenSCs improve survival are likely to be due to the collective effects of their antimicrobial and immunomodulatory properties and tissue regenerative factors expressed by these cells. Moreover, MenSCs in synergy with the antibiotic treatment markedly improved survival in CLP-induced sepsis, suggesting that the synergy between MenSCs and antibiotics improves the sepsis outcome by acting on multiple targets. It is important to note that the implications of the present findings are not exclusively limited to sepsis, as the observed synergy could also potentiate the individual effect of each component in a number of other bacterial infections and subsequently play a pivotal role in the field of infectious diseases.

## Additional files

Additional file 1: Figure S1. Characterization of MenSCs and BMSCs. Stem cells isolated from the menstrual fluid and bone marrow were characterized according to International Society for Cellular Therapy guidelines. **a** MenSCs and BMSCs showed stem cell-like immunophenotypic markers. Fluorescence-activated cell sorting profile of representative cell samples. Blue and orange filled histograms denote the fluorescent profile of the indicated antigens in MenSCs and BMSCs, respectively; red histograms correspond to isotype-matched controls. **b** MenSCs and BMSCs displayed mesodermal differentiation. Tri-lineage differentiation of representative MenSCs and BMSC samples. Cells were cultured with adipogenic, osteogenic, and chondrogenic induction media for 14–21 days and then stained with Oil Red O, Alizarin Red, and Safranin O staining, respectively. Scale bar: 200 μm. *BMSCs* bone marrow-derived mesenchymal stem cells,*MenSCs* menstrual derived mesenchymal stem cells. (PDF 489 kb) Additional file 2: Figure S2. Experimental design for the *in vivo* studies. Diagram of the experimental design of **(a)** survival studies and **(b)** therapeutic effects studies of MenSC administration in mice with CLP-induced sepsis. *AB* antibiotics, *CFU* colony-forming unit, *CLP* cecal ligation and puncture, *CM* conditioned medium, *H&E* hematoxylin and eosin, *MenSCs* menstrual derived mesenchymal stem cells. (PDF 370 kb) Additional file 3: Figure S3. Effect of MenSCs treatment in mice with polymicrobial sepsis. Serum was isolated 24 hours after sepsis induction and administration of different treatments with AB or MenSCs or both (sham, n = 3; saline, n = 5; AB, n = 2–4; MenSCs, n = 4; MenSCs + AB, n = 4) to determine the concentrations of alkaline phosphatase (ALP) (*left panel*) and albumin (*right panel*). Dot plots represent individual values, horizontal bars represent mean values, and vertical bars represent standard error values. ***P* ≤ 0.01. *AB* antibiotics, *MenSCs* menstrual derived mesenchymal stem cells, *ns* not significant. (PDF 180 kb) Additional file 4: Figure S4. Effect of MenSCs treatment on CD4^+^ and CD8^+^ lymphocytes in mice with polymicrobial sepsis. Blood samples were obtained at different time points after induction of sepsis and administration of various treatments to determine specific lymphocyte subsets using flow cytometry (sham, n = 3–5; saline, n = 3–10; AB, n = 3–6; MenSCs, n = 3–6; MenSCs + AB, n = 3–5). Graphs show the percentages of CD45^+^ CD3^+^ CD4^+^ and CD45^+^ CD3^+^ CD8^+^ lymphocytes and the ratios of CD4^+^/CD8^+^ at **(a)** 24 hours and **(b)** 40 hours in untreated and treated mice. Dot plots represent individual values, horizontal bars represent mean values, and vertical bars represent standard error values. **P* ≤ 0.05. *AB* antibiotics, *MenSCs* menstrual derived mesenchymal stem cells, *ns* not significant. (PDF 238 kb)

## Abbreviations

AB: Antibiotics
ALP: Alkaline phosphatase
ALT: Alanine aminotransferase
AMP: Anti-microbial peptide
AST: Aspartate aminotransferase
BMSC: Bone marrow-derived mesenchymal stem cell
BSA: Bovine serum albumin
BUN: Blood urea nitrogen
CFU: Colony-forming unit
CLP: Cecal ligation and puncture
CM: Conditioned medium
DMEM: Dulbecco’s modified Eagle’s medium
FACS: Fluorescence-activated cell sorting
FBS: Fetal bovine serum
IL: Interleukin
MCP-1: Monocyte chemotactic protein 1
MenSC: Menstrual derived mesenchymal stem cell
MSC: Mesenchymal stem cell
PBS: Phosphate-buffered saline
TNF-α: Tumor necrosis factor-alpha
