# Supplementary material for: Combination therapy of menstrual derived mesenchymal stem cells and antibiotics ameliorates survival in sepsis
Source: Stem Cell Res Ther. 2015 Oct 16;6:199. doi: 10.1186/s13287-015-0192-0 (PMC4609164; doi:10.1186/s13287-015-0192-0)
Supplement: Additional file 1: Figure S1. — Characterization of MenSCs and BMSCs. Stem cells isolated from the menstrual fluid and bone marrow were characterized according to International Society for Cellular Therapy guidelines. a MenSCs and BMSCs showed stem cell-like immunophenotypic markers. Fluorescence-activated cell sorting profile of representative cell samples. Blue and orange filled histograms denote the fluorescent profile of the indicated antigens in MenSCs and BMSCs, respectively; red histograms correspond to isotype-matched controls. b MenSCs and BMSCs displayed mesodermal differentiation. Tri-lineage differentiation of representative MenSCs and BMSC samples. Cells were cultured with adipogenic, osteogenic, and chondrogenic induction media for 14–21 days and then stained with Oil Red O, Alizarin Red, and Safranin O staining, respectively. Scale bar: 200 μm. BMSCs bone marrow-derived mesenchymal stem cells,MenSCs menstrual derived mesenchymal stem cells. (PDF 489 kb) [file 13287_2015_192_MOESM1_ESM.pdf]

Additional File 1

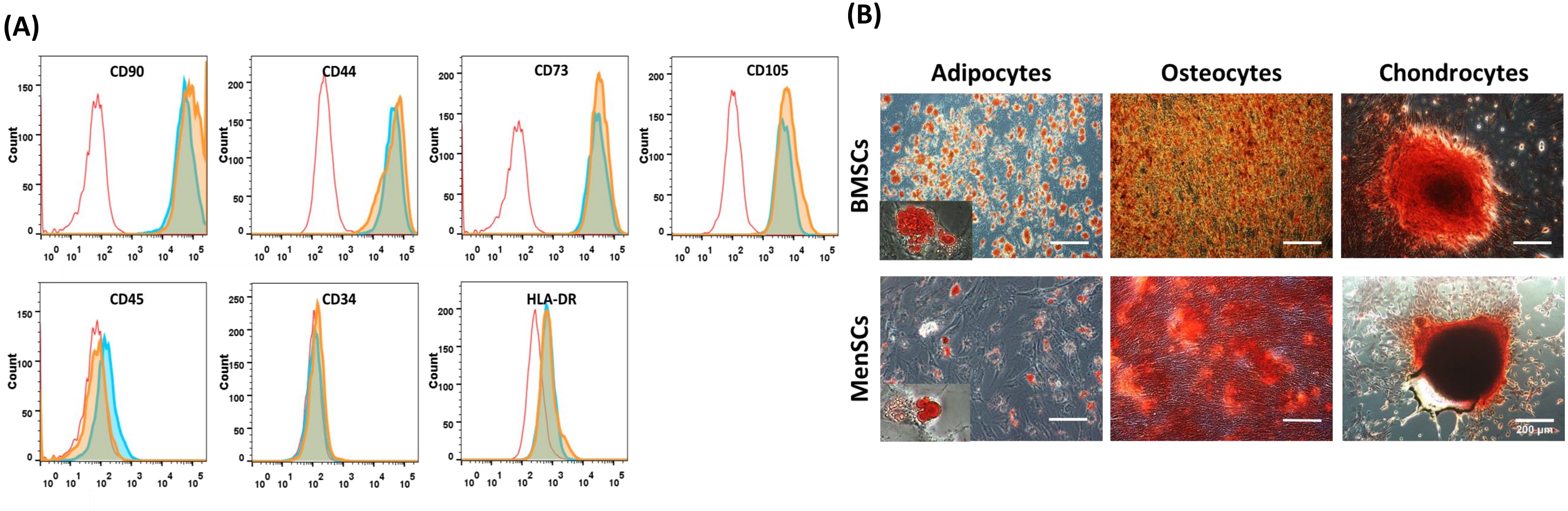

Supplementary Figure 1. Characterization of MenSCs and BMSCs. Stem cells isolated from the menstrual fluid and bone marrow were characterized according to ISCT guidelines. **(A) MenSCs and BMSCs showed stem cell-like immunophenotypic markers**. FACS profile of a representative cells samples. Blue and orange filled histograms denote the fluorescent profile of the indicated antigens in MenSCs and BMSCs, respectively; red histograms correspond to isotype-matched controls. **(B) MenSCs and BMSCs displayed mesodermal differentiation**. Tri-lineage differentiation of a representative MenSCs and BMSCs samples. Cells were cultured with adipogenic, osteogenic and chondrogenic induction media for 14-21 days and then stained with Oil Red O, Alizarin Red and Safranin O staining, respectively. Scale Bar: 200µm.
