# Supplementary material for: Combination therapy of menstrual derived mesenchymal stem cells and antibiotics ameliorates survival in sepsis
Source: Stem Cell Res Ther. 2015 Oct 16;6:199. doi: 10.1186/s13287-015-0192-0 (PMC4609164; doi:10.1186/s13287-015-0192-0)
Supplement: Additional file 2: Figure S2. — Experimental design for the in vivo studies. Diagram of the experimental design of (a) survival studies and (b) therapeutic effects studies of MenSC administration in mice with CLP-induced sepsis. AB antibiotics, CFU colony-forming unit, CLP cecal ligation and puncture, CM conditioned medium, H&E hematoxylin and eosin, MenSCs menstrual derived mesenchymal stem cells. (PDF 370 kb) [file 13287_2015_192_MOESM2_ESM.pdf]

Additional File 2

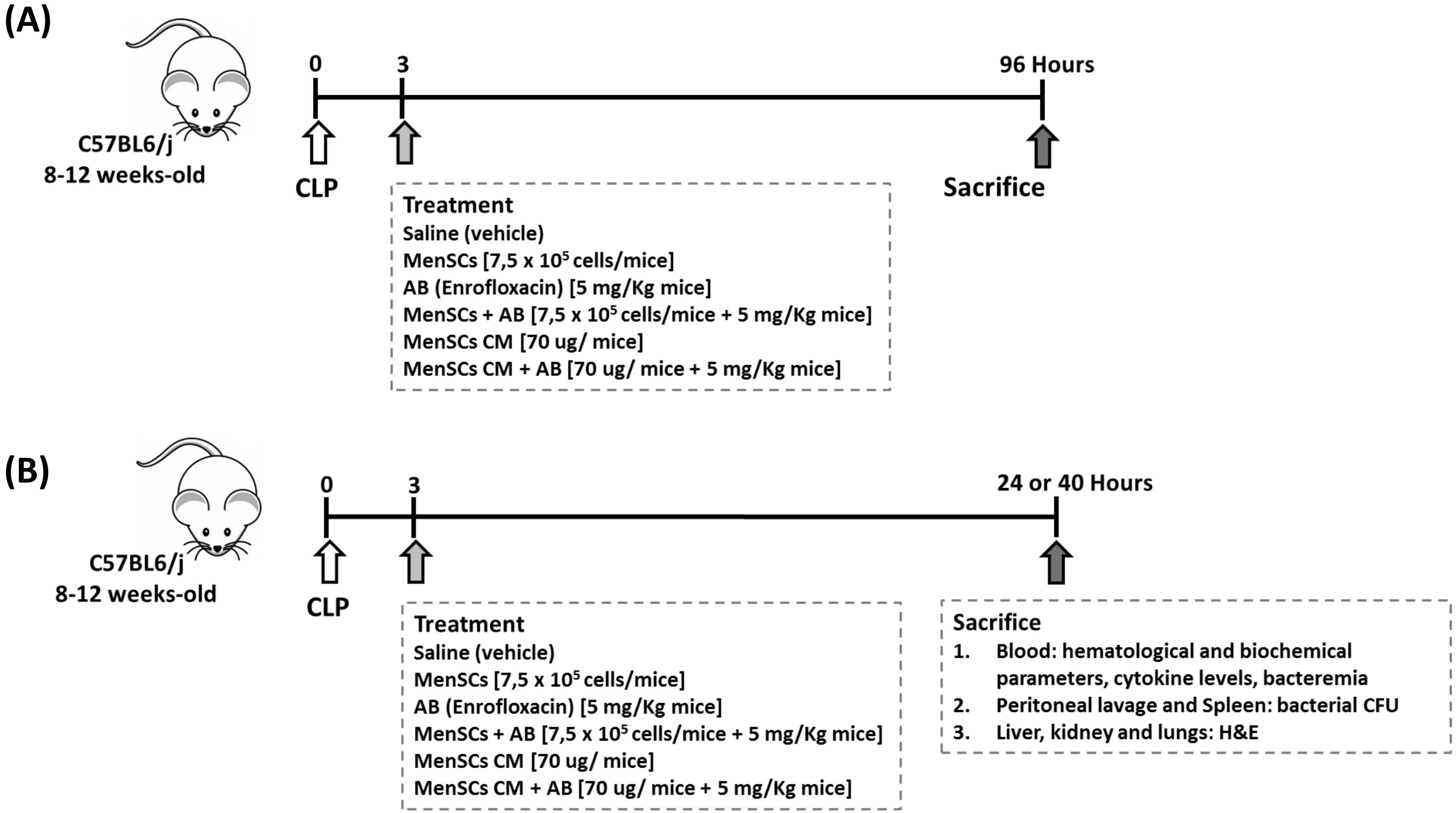

Supplementary Figure 2. Experimental design for the *in vivo* studies. Diagram of the experimental design of **(A)** survival studies and **(B)** therapeutical effects studies of MenSCs administration in mice with CLP-induced sepsis. Abbreviations: MenSCs, menstrual derived mesenchymal stem cells; AB, antibiotics; CM, conditioned medium; CFU, colony forming units.
