# Supplementary material for: Combination therapy of menstrual derived mesenchymal stem cells and antibiotics ameliorates survival in sepsis
Source: Stem Cell Res Ther. 2015 Oct 16;6:199. doi: 10.1186/s13287-015-0192-0 (PMC4609164; doi:10.1186/s13287-015-0192-0)
Supplement: Additional file 3: Figure S3. — Effect of MenSCs treatment in mice with polymicrobial sepsis. Serum was isolated 24 hours after sepsis induction and administration of different treatments with AB or MenSCs or both (sham, n = 3; saline, n = 5; AB, n = 2–4; MenSCs, n = 4; MenSCs + AB, n = 4) to determine the concentrations of alkaline phosphatase (ALP) (left panel) and albumin (right panel). Dot plots represent individual values, horizontal bars represent mean values, and vertical bars represent standard error values. **P ≤ 0.01. AB antibiotics, MenSCs menstrual derived mesenchymal stem cells, ns not significant. (PDF 180 kb) [file 13287_2015_192_MOESM3_ESM.pdf]

Additional File 3

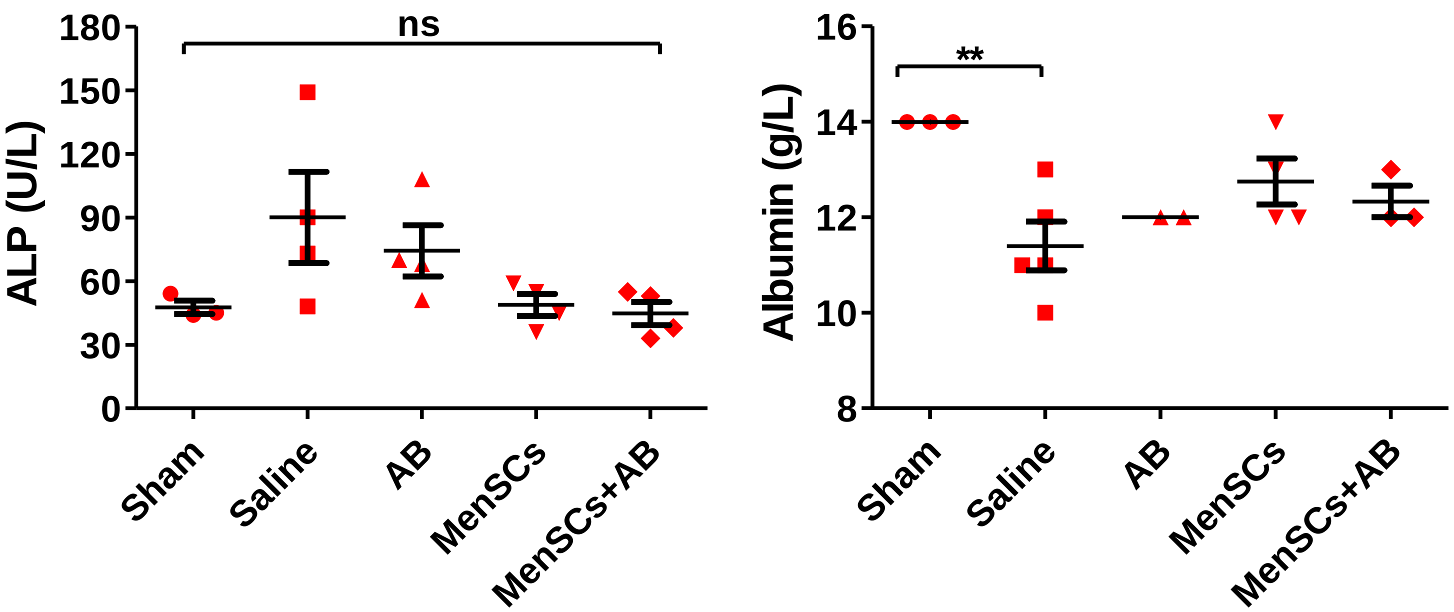

Supplementary Figure 3. Effect of MenSCs treatment in mice with polymicrobial sepsis. Serum was isolated 24 hours post sepsis induction and administration of different treatments with antibiotics (AB) and/or MenSCs (Sham, n=3; Saline, n=5; AB, n=2-4; MenSCs, n=4; MenSCs+AB, n=4) to determine the concentrations of alkaline phosphatase (ALP) (left panel) and albumin (right panel). Dot plots represent individual values, horizontal bars represent mean values and vertical bars represent SE values. ** $p \leq 0.01$. Abbreviations: MenSCs, menstrual derived mesenchymal stem cells; AB, antibiotics; SE, standard error; ns, not significant.
