# Supplementary material for: Combination therapy of menstrual derived mesenchymal stem cells and antibiotics ameliorates survival in sepsis
Source: Stem Cell Res Ther. 2015 Oct 16;6:199. doi: 10.1186/s13287-015-0192-0 (PMC4609164; doi:10.1186/s13287-015-0192-0)
Supplement: Additional file 4: Figure S4. — Effect of MenSCs treatment on CD4+ and CD8+ lymphocytes in mice with polymicrobial sepsis. Blood samples were obtained at different time points after induction of sepsis and administration of various treatments to determine specific lymphocyte subsets using flow cytometry (sham, n = 3–5; saline, n = 3–10; AB, n = 3–6; MenSCs, n = 3–6; MenSCs + AB, n = 3–5). Graphs show the percentages of CD45+ CD3+ CD4+ and CD45+ CD3+ CD8+ lymphocytes and the ratios of CD4+/CD8+ at (a) 24 hours and (b) 40 hours in untreated and treated mice. Dot plots represent individual values, horizontal bars represent mean values, and vertical bars represent standard error values. *P ≤ 0.05. AB antibiotics, MenSCs menstrual derived mesenchymal stem cells, ns not significant. (PDF 238 kb) [file 13287_2015_192_MOESM4_ESM.pdf]

Additional File 4

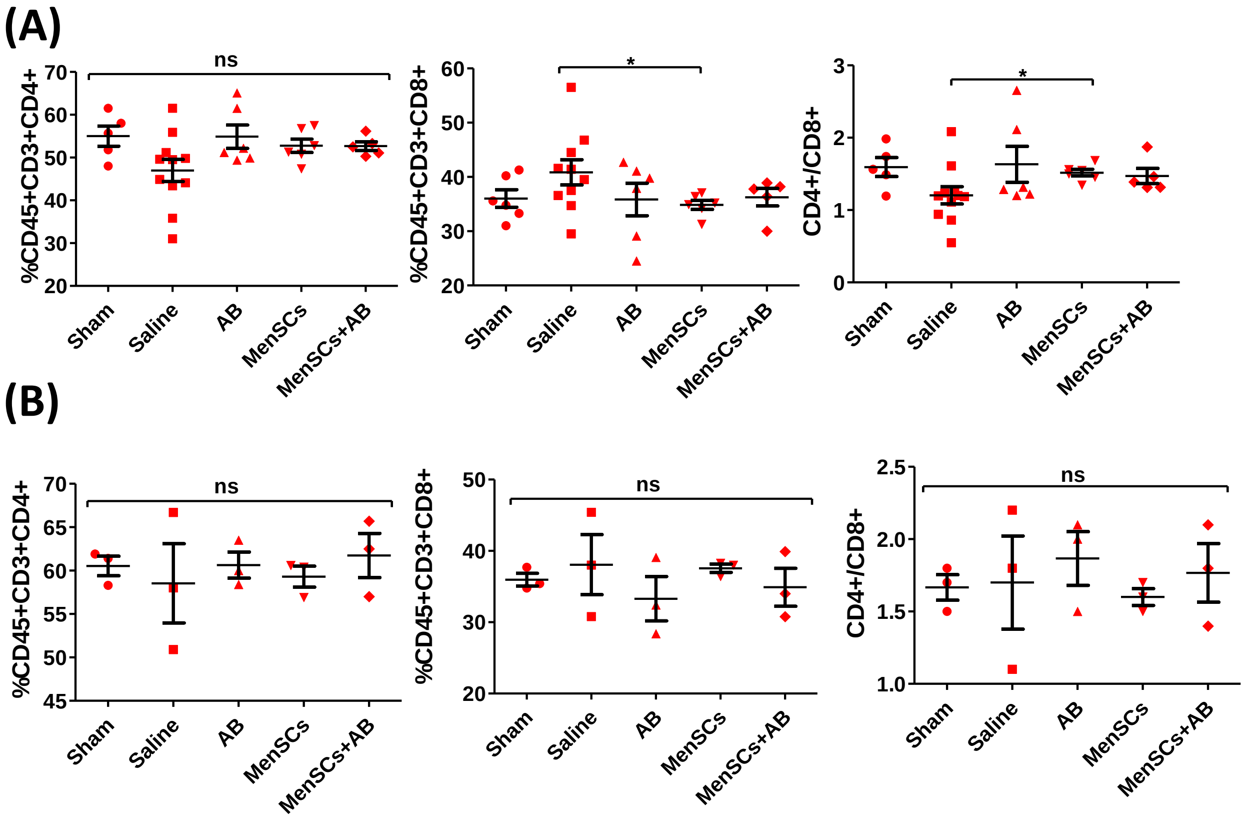

Figure S4. Effect of MenSCs treatment on CD4+ and CD8+ lymphocytes in mice with polymicrobial sepsis. Blood samples were obtained at different time points after induction of sepsis and administration of various treatments to determine specific lymphocyte subsets using flow cytometry (Sham, n=3-5; Saline, n=3-10; AB, n=3-6; MenSCs, n=3-6; MenSCs + AB, n=3-5). Graphs show the percentages of CD45+CD3+CD4+ and CD45+CD3+CD8+ lymphocytes, and the ratios of CD4+/CD8+ at **(A)** 24 hours and **(B)** 40 hours in untreated and treated mice. Dot plots represent individual values, horizontal bars represent mean values and vertical bars represent SE values. * p ≤ .05. Abbreviations: MenSCs, menstrual derived mesenchymal stem cells; AB, antibiotics; SE, standard error; ns, not significant.
